# Simultaneous Discrimination of Cys/Hcy and GSH With Simple Fluorescent Probe Under a Single-Wavelength Excitation and its Application in Living Cells, Tumor Tissues, and Zebrafish

**DOI:** 10.3389/fchem.2022.856994

**Published:** 2022-03-11

**Authors:** Dongling Yan, Likun Liu, Xiangbao Liu, Qi Liu, Peng Hou, Hao Wang, Chunhui Xia, Gang Li, Chunhui Ma, Song Chen

**Affiliations:** ^1^ College of Pharmacy, Qiqihar Medical University, Qiqihar, China; ^2^ Research Institute of Medicine & Pharmacy, Qiqihar Medical University, Qiqihar, China

**Keywords:** fluorescent probe, single-wavelength excitation, tumor tissues, thiols, cell imaging

## Abstract

Owing to the important physiological sits of biothiols (Cys, Hcy, and GSH), developing accurate detection methods capable of qualitative and quantitative analysis of biothiols in living systems is needed for understanding the biological profile of biothiols. In this work, we have designed and synthesized a 4′-hydroxy-[1,1′-biphenyl]-4-carbonitrile modified with NBD group-based fluorescent probe, BPN-NBD, for sensitive detection of Cys/Hcy and GSH by dual emission signals via a single-wavelength excitation. BPN**-**NBD exhibited an obvious blue fluorescence (λ_max_em = 475 nm) upon the treatment with GSH and reacted with Cys/Hcy to give a mixed blue-green fluorescence (λ_max_em = 475 and 545 nm). Meanwhile, BPN-NDB performed sufficient selectivity, rapid detection (150 s), high sensitivity (0.011 µM for Cys, 0.015 µM for Hcy, and 0.003 µM for GSH) and could work via a single-wavelength excitation to analytes and had the ability to image Cys/Hcy from GSH in living MCF-7 cells, tumor tissues, and zebrafish by exhibiting different fluorescence signals. Overall, this work provided a powerful tool for thiols visualization in biological and medical applications.

## Introduction

Amino acids are presently found in living organisms to make generous contributions in physiological and pathological processes, which are structural pillars of proteins and necessary nutrients for human beings involved in anti-aging, bone growth, immune regulation, and metabolism (Ball et al., 2006; Hu et al., 2019; Ren et al., 2020a). The fluctuation of such amino acids in content is closely related to serious health problems. Among the various amino acids, biothiols (Cys, Hcy, and GSH) act as the most essential sulfur-containing biological compounds to maintain biological redox homeostasis and have gained considerable attention (dos Santos et al., 2020; Sun et al., 2021). Cys is a precursor of protein synthesis detected in mammalian cells. The deregulation of Cys is correlated with many syndromes, including retarded growth, skin lesions, neurotoxicity, muscle and fat loss, and hair depigmentation (Liu et al., 2021a; Mei et al., 2021; Pham et al., 2021). Hcy can serve as an indicator for various diseases, such as thrombosis, cardiovascular issues, and neuropsychiatric illness. Hyperhomocysteinemia has been generated with the high levels of Hcy (over 15 μmol/L) in serum (Qi et al., 2021; Su et al., 2021). The range (1–10 mM) of GSH, as a representative nonprotein mercaptan, in cells plays the role of detoxifying antioxidant to protect cells from the reactive oxygen damage of lipid peroxides, free radicals, and heavy metals. However, GSH disorder is observed in both AIDS and cancer (Sun et al., 2020; Liu et al., 2021b). Owing to the important physiological sites of biothiols, developing accurate detection methods capable of qualitative and quantitative analysis of biothiols in living systems is necessary for understanding the biological profile of biothiols.

Optical probes have been a powerful tool for monitoring and imaging anions, cations, enzymes, and biomolecules *in vitro*/*vivo* because of their easy operation, high sensitivity, good selectivity, and noninvasive detection (Chen et al., 2018a; Hou et al., 2020; Li et al., 2020; Park et al., 2020; Yang et al., 2020; Cui et al., 2021; Du et al., 2021). At present, a huge amount of fluorescent probes have been developed for the investigation of Cys, Hcy, and GSH in living cells based on cyclization with aldehyde, Michael addition, cleavage of sulfonamide, disulfide, selenium–nitrogen, and sulfonate ester (Chen et al., 2018b; Chen et al., 2020; Yue et al., 2020; Chen et al., 2021a; Li et al., 2021a; Zhang et al., 2021a; Zheng et al., 2021; Chen et al., 2022). However, owing to the similar structures and reactivities of GSH and Cys/Hcy, simultaneous selective detection of Cys/Hcy and GSH is still a great challenge. Recently, the nitrobenzoxadiazole (NBD) group, having an excellent sensing moiety, has been used to distinguish Cys/Hcy from GSH with dual emission signals in the construction of optical sensors (Ren et al., 2020b; Chen et al., 2021b; Li et al., 2021b; Zhang et al., 2021b; Rong et al., 2021). Despite these obvious advances, some of the reported NBD-based probes suffer from complex synthesis processes, low sensitivity, and slow response. In addition, most of such fluorescent probes for the discriminative detection of Cys/Hcy and GSH are based on double excitation lights. Compared with them, a single-wavelength excitation fluorescent probe has the characteristics of easy data collection and low background noise in the fluorescence detection process, which greatly improve the accuracy of detection (Chen et al., 2016; Ren et al., 2021). Unfortunately, such a fluorescent probe is still rare. Therefore, the development of simultaneous discrimination of Cys/Hcy and GSH with a simple fluorescent probe under a single-wavelength excitation is highly valuable.

On the basis of the above-mentioned concerns, we have designed and synthesized in this work a simple fluorescent probe, BPN-NBD, which could effectively discriminate Cys/Hcy and GSH with dual emission signals via a single-wavelength excitation. Probe BPN-NBD was constructed by combining two fluorophores [4′-hydroxy-(1,1′-biphenyl)-4-carbonitrile, BPN-OH, and NBD] by a facile ether bond. In the process of detecting, probe BPN-NBD exhibited an obvious blue fluorescence (λ_max_em = 475 nm) with high sensitivity upon the treatment with GSH, while it reacted with Cys/Hcy to give a mixed blue-green fluorescence (λ_max_em = 475 and 545 nm). Compared with previous reports (Supplementary Table S1), BPN-NBD has many advantages such as excellent selectivity, rapid detection ability (150 s), being easy to synthesize (one-step), high sensitivity (0.011 µM for Cys, 0.015 µM for Hcy, 0.003 µM for GSH), and could work via a single-wavelength excitation. Notably, BPN-NBD was successfully applied in imaging of Cys/Hcy from GSH in living MCF-7 cells, tumor tissues, and zebrafish through a dual-emission signal manner. All of the results demonstrated that BPN-NBD could be a powerful tool for biosystem thiols visualization.

## Experimental

### Instruments and Reagents

All nuclear magnetic resonance (NMR) spectra of BPN-NBD were collected by a Bruker Avance 400 MHz spectrometer. A Zeiss LSM710 Wetzlar (German) laser scanning confocal microscope was used for fluorescence imaging. High-resolution mass spectrometry (HRMS) data of new compounds were tested on AB Sciex TripleTOF 4600. UV–vis absorption and fluorescence data were collected through a Shimadzu UV-2450 spectrophotometer and a HITACHI F-4600 fluorescence spectrophotometer, respectively. Ultra-high performance liquid chromatography (UHPLC) analyses were conducted on a Shimadzu NexeraX2 UHPLC LC-30A. Unless otherwise specified, all raw materials used for synthesis were purchased from the chemical suppliers in China and used directly without further refining.

### Syntheses of BPN-NBD

BPN-OH (195 mg, 1.0 mmol) and NBD-Cl (238.8 mg, 1.2 mmol) were dissolved in 30 ml dichloromethane, and then, the Et_3_N (121.3 mg, 1.2 mmol) was added to the above-mentioned solution under stirring. The resulting mixture reacted at room temperature for 4 h. The color of the solution gradually deepened as time progressed. After the reaction was completed, the yellow precipitated was extracted three times with dichloromethane (10 ml/3). Next, the solvent was removed under reduced pressure, and a crude product was produced. Then, the crude product was purified by column chromatography (CH_2_Cl_2_:PE = 1:1) to obtain a light-yellow solid **BPN-NBD** (272.1 mg, 76% yield). ^1^H NMR (400 MHz, DMSO-*d*_*6*_) δ 8.67 (d, J = 8.4 Hz, 1H), 8.00 (d, J = 2.0 Hz, 1H), 7.98 (d, J = 2.0 Hz, 1H), 7.97 (s, 4H), 7.62 – 7.52 (m, 2H), 6.84 (d, J = 8.4 Hz, 1H). ^13^C NMR (101 MHz, DMSO-*d*_*6*_) δ 153.45, 152.87, 145.43, 144.40, 143.33, 136.75, 135.43, 132.93, 130.51, 129.52, 127.65, 121.48, 118.76, 110.26. HRMS (ESI) m/z: calcd for [C_19_H_11_N_4_O_4_]^+^ 359.0739, found 359.0780.

### Procedure for Optical Data Measurements

The 10.0 mM source of analytes (amino acids and ions) was freshly prepared by being dissolved in twice-distilled water. Fluorescence spectra were measured in 7.4 20 mM PBS (containing 1.0 mM CTAB), in which a certain amount of biothiols (Cys, Hcy, or GSH) standard solution (3.0 mM) and other competitive analytes reacted with BPN-NBD in a 3.0 ml quartz cuvette. The testing solution was completely incubated at ambient conditions within a short time (about 150 s) to record. The fluorescence spectra were obtained with the excitation wavelength at 365 nm and 5.0/5.0 nm for slit width.

### Cell Culture and Imaging

The MCF-7 cells were used to perform the fluorescent imaging of BPN-NBD. The cytotoxicity of BPN-NBD was evaluated by MCF-7 cells cultured in 96-well plates and placed in a medium with different BPN-NBD concentrations (0.0–100.0 µM) for another 24 h. The MTT reagent was added to each well of the plate to obtain data of absorbance measurement at 490 nm for testing cell viability. As considerable previous literature described, the MCF-7 cells were grown on glass-bottom dishes in Dulbecco’s Modified Eagle’s Medium with a humidified atmosphere of 5% CO_2_ overnight for cell attachment. For imaging experiments, the MCF-7 cells were first seeded in probe BPN-NBD (10.0 μM) for 30 min and then washed with PBS prior to imaging. The adhered MCF-7 cells, as control experiments, were pre-treated with thiol scavenger N-ethylmaleimide (NEM, 1.0 mM) for 30 min, and fluorescence image was acquired by incubated in probe BPN-NBD (10.0 μM) for another 30 min. In addition, NEM**-**stained MCF-7 cells were treated with biothiol (150.0 μM Cys, Hcy, or GSH) for 30 min and probe BPN-NBD (10.0 μM) for another 30 min. Before the imaging, all the abovementioned dyed MCF-7 cells were washed three times with PBS and recorded with a confocal fluorescence microscope.

### Zebrafish Imaging

To validate the biological potential of BPN-NBD as a fluorescent scaffold, fluorescence imagings toward biothiols in living zebrafish were further explored. Three-day-old zebrafish were obtained from Eze-Rinka Company (Nanjing, China). Five groups were operated in different ways. For the first group, the zebrafish were stained with BPN-NBD alone for 30 min. For visualizing biothiol-induced fluorescence, the other test groups were all pre-treated with NEM. The second test group was then given an incubation of BPN-NBD for 30 min, while the remaining three experimental groups were incubated with biothiol (Cys, Hcy, or GSH, respectively) for 30 min followed by treatment with BPN-NBD for another 30 min. After the excess BPN-NBD adopted with PBS flushing solution was removed, the zebrafish fluorescence imagings were captured by a confocal microscope at green and blue channels.

### Tumor Tissue Imaging

The Balb/c mice (female) were purchased from Liaoning Changsheng biotechnology Co., Ltd. The animals were then evaluated using the protocols approved by the Animal Ethics Committee of Qiqihar Medical University. Nine female Balb/c mice at 4 weeks of age were divided into three groups (groups A, B, and C). Next, 0.1 ml of a 4T1 cell suspension containing 5 × 106 cells was injected into the left armpit of each mouse. When the tumors reached approximately 0.8 cm in diameter, the experiments were performed (induced by tail vein injection of 200.0 μM Cys(A)/GSH(B) for 1.5 h and 50.0 μM BPN-NBD for another 1.5 h). The Balb/c mice of the control experiment (C) were individually injected with BPN-NBD (50.0 μM) for 1.5 h. After the mice were anesthetized with sodium pentobarbital anesthetic administered, the tumor tissue was harvested from these mice for confocal fluorescence imaging. Fresh tumor tissues were fixed in 4% paraformaldehyde solution for over 48 h. The tissue was cut into a suitable tissue block (nearly 5 mm), placed in a tissue-embedded box, and dehydrated in a Leica ASP200S automatic vacuum dehydrator. The preparation and seal of tissue slices with a section thickness of 4 μm were operated in the conventional processing (Wan et al., 2021).

## Results and Discussion

### Design and Synthesis

**SCHEME 1:**
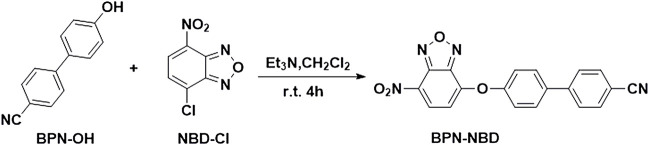
The synthesis of BPN-NBD.

**SCHEME 2:**
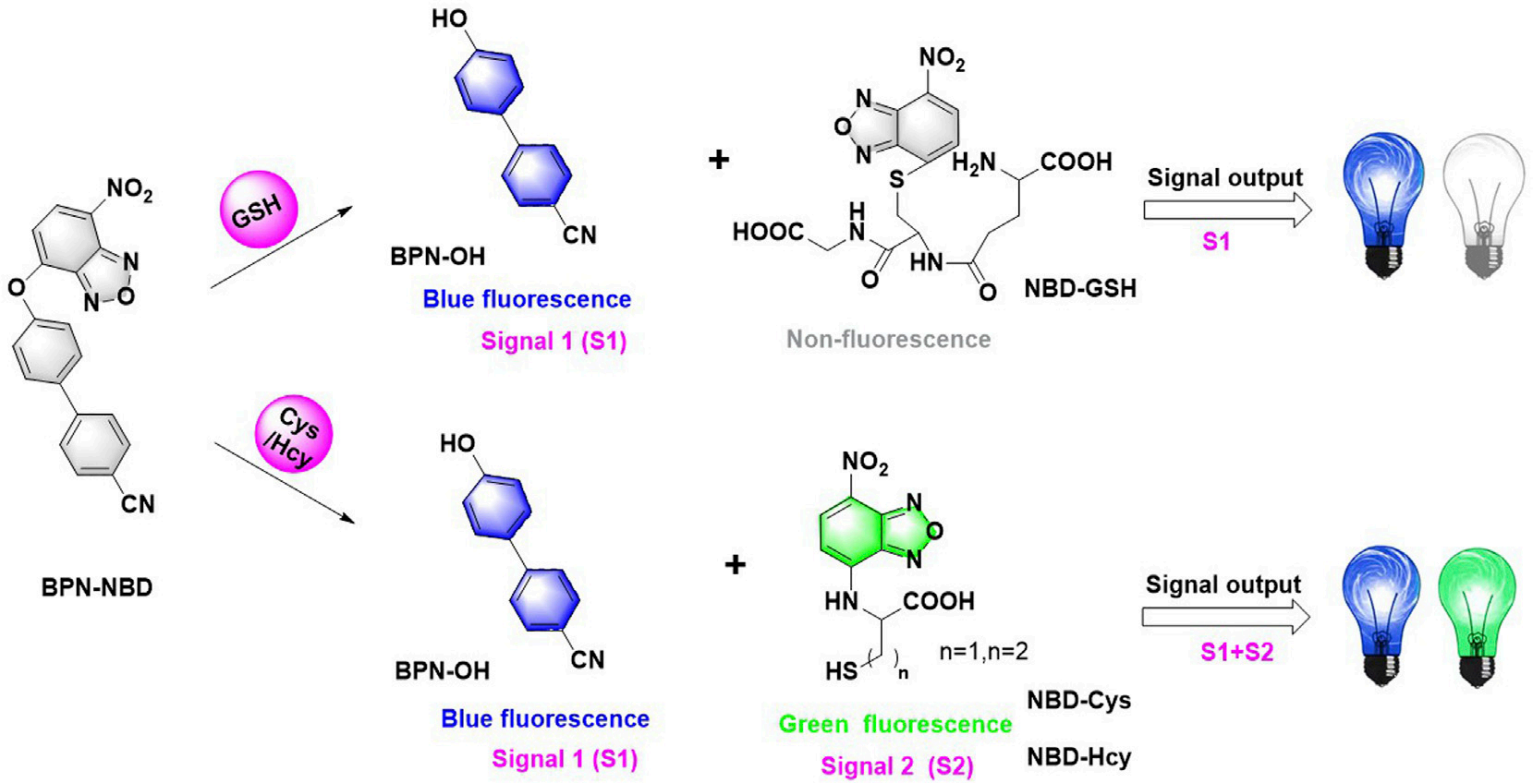
The design strategy for BPN-NBD.

The molecular structure of BPN-NBD was designed based on the following considerations. 4′-hydroxy-[1,1′-biphenyl]-4-carbonitrile, BPN-OH, was selected as the chromophore because of its good biocompatibility, intense luminescence, large Stokes shift, and brilliant photostability (Chen et al., 2018c). A literature survey indicated that the NBD group could function not only as the potential green fluorophore but also as an outstanding response site for Cys/Hcy and GSH (Jiang et al., 2021; Yang et al., 2021). On the basis of the above-mentioned concerns, two fragments (BPN-OH and NBD) were fused into one molecule to develop a new 4′-hydroxy-[1,1′-biphenyl]-4- carbonitrile-based fluorescent probe BPN-NBD. The design rationale was illustrated in Scheme 2. BPN-NBD was nearly nonfluorescent in the blue and green region because of the PET process ascribed to the NBD group. However, the NBD moiety in probe BPN-NBD was attacked by Cys/Hcy via nucleophilic substitution reaction when BPN-NBD reacted with Cys/Hcy. Subsequently, a strong blue fluorescent product BPN-OH and the Smile rearrangement product (green fluorescence) NBD-Cys/Hcy were formed. When BPN-NBD encountered GSH, BPN-NBD only output the brilliant fluorescence in the blue region derived from chromophore BPN-OH. These properties enabled BPN-NBD to be used for the detection of Cys/Hcy from GSH through a dual-emission signal manner. Probe BPN-NBD was synthesized by simple one-step (Scheme 1), and the corresponding characterization data (HRMS, ^13^C NMR, and ^1^H NMR) of BPN-NBD were depicted in the Supporting Information (Supplementary Figures S15–S17).

### Spectral Response

**FIGURE 1:**
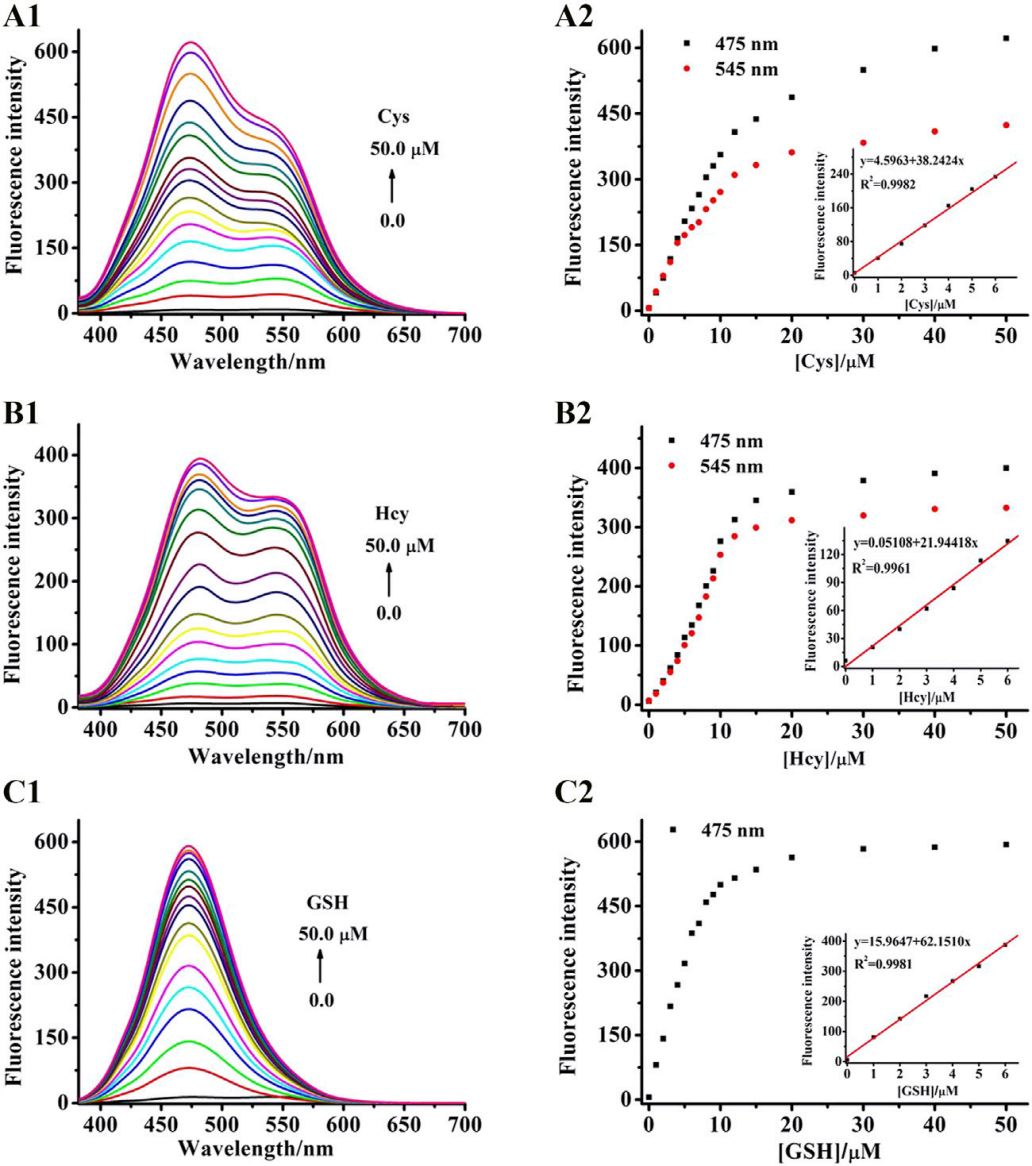
**(A1–C1)** Fluorescence response of BPN-NBD different Cys/Hcy and GSH concentrations in the 0.0–50.0 μM range (pH 7.4 PBS buffer), respectively. **(A2–C2)** Calibration curve of BPN-NBD vs. the concentrations of Cys/Hcy or GSH, and the linear relationship between fluorescence intensity at 475 nm of BPN-NBD (10.0 μM) and Cys/Hcy or GSH (0.0–6.0 μM).

With BPN-NBD in hand, the fluorescence spectral properties of BPN-NBD (10.0 µM) were measured in pH 7.4 20 mM PBS (containing 1.0 mM CTAB) with varying concentrations (0.0–50.0 µM) of three biothiols (Cys, Hcy, and GSH) under a single-wavelength excitation at 365 nm. As seen in Figure 1, free BPN-NBD (10.0 µM) initially exhibited no fluorescence. However, after Cys/Hcy (Figures 1A1,B1) was added to probe BPN-NB**D** (10.0 µM), a concentration-dependent fluorescence emission spectra increment appeared at two-emission fluorescence of 475 and 545 nm (approximately 80-fold and 120-fold increase for 5 equiv. of Cys and Hcy, respectively). Compared with Cys/Hcy, GSH (50.0 µM) directly reacting with BPN-NBD (10.0 µM) only triggered a remarkable fluorescence enhancement at 475 nm (up to about 120-fold) (Figure 1C1). The results indicated that there were good linearities between the emission intensities and Cys/Hcy (Figures 1A2,B2) or GSH (Figure 1C2) content in the range of 0.0–6.0 µM (*R* ^2^ = 0.9982, 0.9981, 0.9961). Based on the equation of intensities as the amount, the limit of detection concentration (LOD) of BPN-NBD was calculated (0.011 µM for Cys, 0.015 µM for Hcy, 0.003 µM for GSH, respectively), which are comparable with previously reported results (Supplementary Table S1). The abovementioned data demonstrated that BPN-NBD had excellent sensitivity for quantitative analysis toward Cys/Hcy and GSH under the experimental conditions.

### Selectivity Study

**FIGURE 2:**
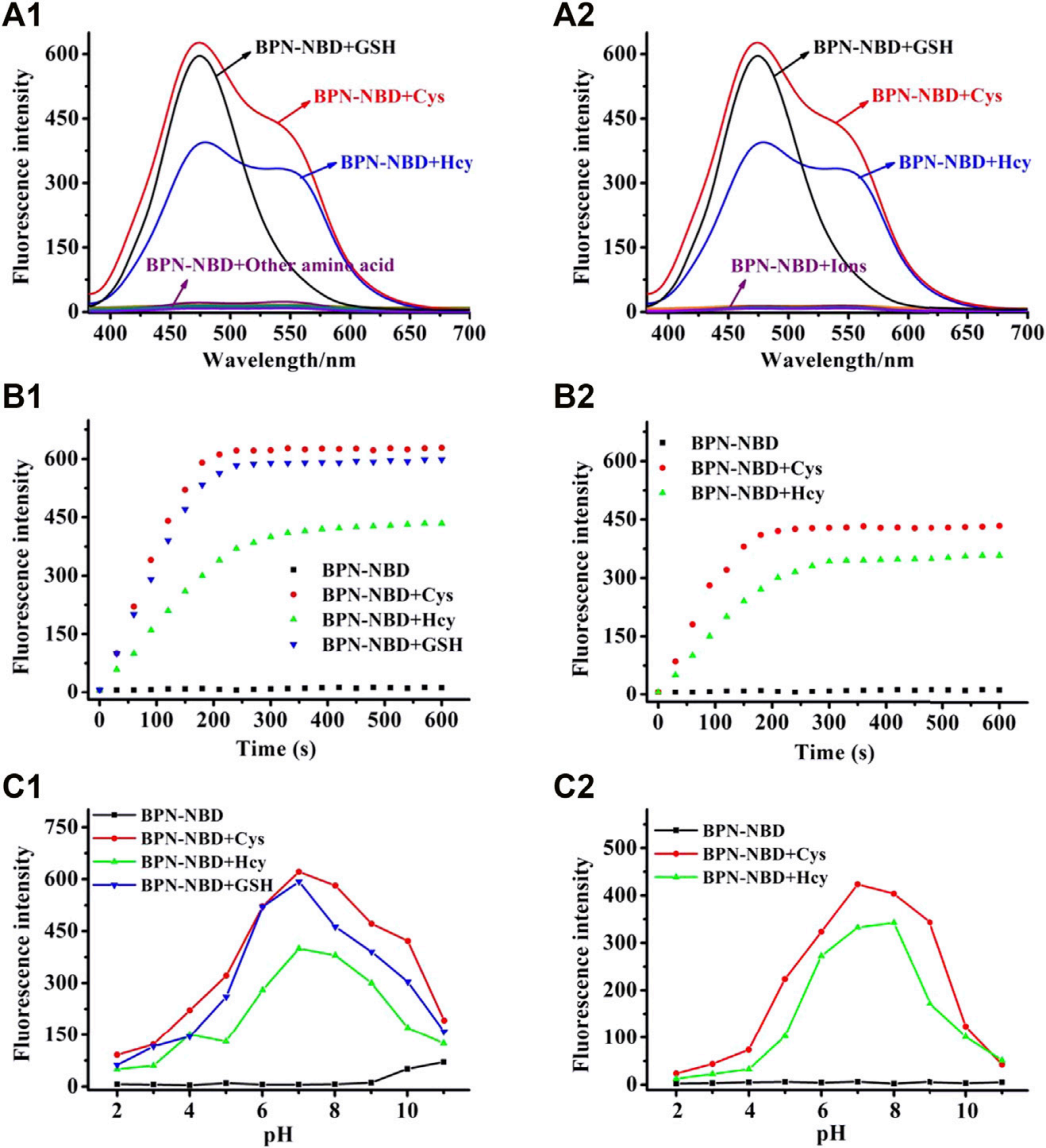
**(A1,A2)** Fluorescence spectral changes of BPN-NBD (10.0 μM) for (50.0 μM) biothiols in pH 7.4 PBS buffer (Cys/Hcy and GSH) or other analytes (100.0 μM for other amino acids, 500.0 μM for ions). **(B1,B2)** Photostability of BPN-NBD (10.0 μM) when reacted with Cys/Hcy or GSH (50.0 μM) over time-dependent fluorescence intensity changes at 475 and 545 nm in pH 7.4 PBS buffer. **(C1,C2)** Effect of pH (2.0–11.0) on BPN-NBD (10.0 μM) in the absence/presence of Cys/Hcy or GSH (50.0 μM) at 475 and 545 nm.

To inspect the availability of BPN-NBD toward biothiols with high specificity, fluorescent probe BPN-NBD (10.0 µM) related to other potentially competitive species was investigated in pH 7.4 20 mM PBS (containing 1.0 mM CTAB). As depicted in Figure 2A1, only the three biothiols (Cys, Hcy, and GSH) displayed significant fluorescence increases in BPN-NBD (10.0 µM) detection system, whereas the fluorescence responses barely varied with other amino acids (including Leu, Ser, Ile, Asp, Ala, Val, Pro, Gly, Met, Phe, His, Trp, Arg, Tyr, Glu, and Thr) at 475 nm. The great selectivity of BPN-NBD (10.0 µM) toward biothiols (Figure 2A2) was also found compared with potential biologically relevant ions (including F^−^, Cl^−^, SO_4_^2−^, SO_3_^2−^, NO_3_^−^, AcO^−^, K^+^, Na^+^, Al^3+^, Mg^2+^, Zn^2+^, Cu^2+^, Mn^2+^, Fe^2+^, Fe^3+^, and Ca^2+^), and a nearly imperceptible change was observed for even ions employed at much higher concentration (500.0 µM) at 475 nm. Noticeably, when Cys/Hcy (100.0 µM) was added to BPN-NBD (10.0 µM) system, the fluorescence at 545 nm was also lit up at the same time differently from that of GSH.1 Furthermore, the coexistence experiments in the presence of biothiols with the competitive analytes were performed (Supplementary Figures S2–S11). As expected, exposing BPN-NBD to the mixture of abovementioned analytes and biothiols rendered similar results to the case of biothiols only. The coexistence of the competitive analytes did not cause interference in the analysis of biothiols. These observations clearly proved that BPN-NBD not only responded to Cys/Hcy or GSH selectively against other related substances but also discriminated Cys/Hcy from GSH in aqueous media.

### Kinetic and pH Study

Response time is an important index for reaction-based fluorescence probes. Thus, the dynamic reaction of BPN-NBD incubated with different biothiols (Cys, Hcy, and GSH) was acquired to evaluate the time-dependent fluorescence response. As shown in Figures 2B1,B2, BPN-NBD (10.0 µM) held stable emission signal output (at 475/545 nm) with the extended response time. When BPN-NBD mixed with the biothiols (50.0 µM), we found the dramatic spectrum increase reached a plateau state within 150 s for three states (BPN-NBD upon addition of Cys/Hcy or GSH) at 475 nm. Similar results of the fluorescence spectrum of BPN-NBD with Cys/Hcy emerged at the emission wavelength of 545 nm, which indicated that BPN-NBD could be used as an effective candidate for real-time sensing biothiols. The pH effect on probe BPN-NBD (10.0 µM) with and without biothiols (50.0 µM) was evaluated (ranging from 2 to 11) (Figures 2C1,C2) to verify the sensing property of BPN-NBD for biothiols under physiological environment. In the absence of biothiols, no remarkable intensity change was observed. BPN-NBD exhibited excellent stability under a wide range of pH conditions. In the case of Cys/Hcy and GSH, the strong fluorescence augmentation of BPN-NBD appeared with altering pH (5.0–9.0), whereas a weak fluorescence behavior occurred when the pH range was 2.0–4.0 or over 10. The optimal range of pH values was 6.0–8.0 at 475 nm or 545 nm, which meant BPN-NBD could appreciably monitor biothiols with a pH value around 7.4. These results implied that BPN-NBD had a latent capability for biological applications in the typical physiological conditions.

### Mechanism Studies

**FIGURE 3:**
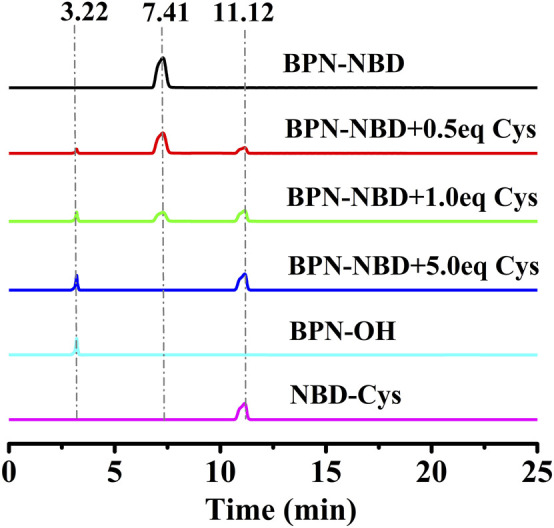
The UHPLC chromatograms: (black) BPN-NBD; (red/green/blue) BPN-NBD with gradually increasing amount (0.5, 1.0, 5.0) equiv. of Cys incubated for 3 min; (cyan) BPN-OH; (magenta) NBD-Cys. Condition: eluent, H_2_O/CH_3_CN (v/v, 4/6), flow rate, 0.3 ml/min; temperature, 25°C; injection volume, 10.0 μL.

The HRMS spectra of BPN-NBD + Cys and BPN-NBD + GSH were checked first to verify the sensing mechanism of BPN-NBD toward Cys/Hcy and GSH. As displayed in Supplementary Figures S12, S13, a mass spectral peak at m/z 285.0263 corresponding to [M + H^+^]^+^ for NBD-Cys and a peak at m/z 196.0744 corresponding to [M + H^+^]^+^ for BPN-OH were represented in the mixture of Cys with BPN-NBD. The desired products (m/z 471.0886 for NBD-GSH and m/z 196.0750 for BPN-OH) were also obtained in the mixed solution of BPN-NBD and GSH. UHPLC was used to obtain insights into the corresponding sensing mechanism. As displayed in Figure 3, BPN-NBD exhibited a main peak with a retention time of 7.41 min. However, two new peaks with retention times of 3.22 and 11.12 min emerged when BPN-NBD encountered Cys, and the two new peaks were further confirmed to be BPN-OH and NBD-Cys, respectively. The data powerfully support the proposed response mechanism in Scheme 2.

### Imaging of Thiols in Living Cells

**FIGURE 4:**
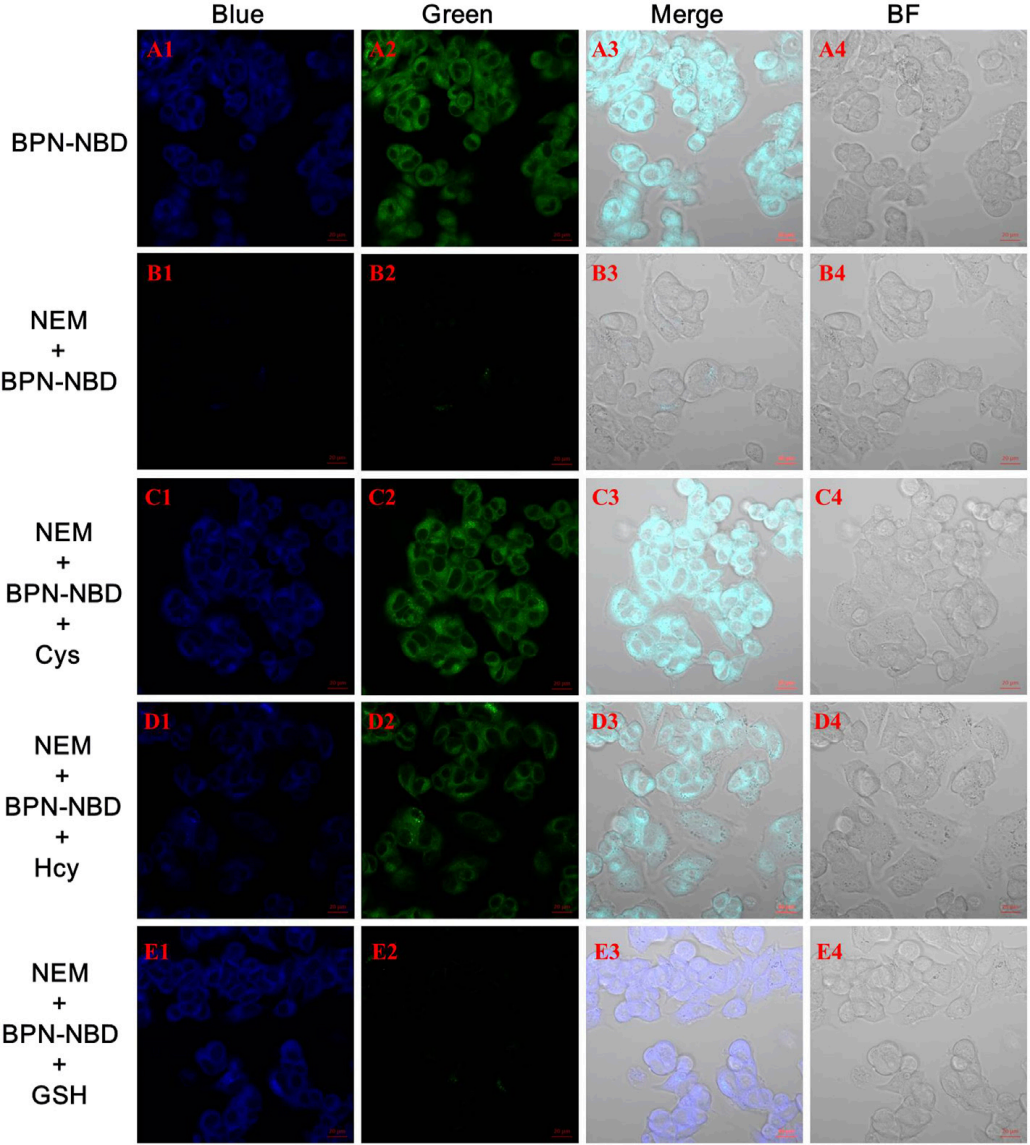
The Bright-field and fluorescence confocal imaging of biothiols in MCF-7 cells with BPN-NBD. **(A)** MCF-7 cells stained only with BPN-NBD (10.0 µM) for 30 min **(B)** NEM-MCF-7-cells incubated with BPN-NBD (10.0 µM) for 30 min **(C–E)** The similar cultivations for NEM-MCF-7-cells treated with the replacement of Cys **(C)** /Hcy **(D)** or GSH **(E)** (150.0 µM) for 30 min and then incubated with BPN-NBD (10.0 µM) for another 30 min. Emissions were collected at 425–475 nm for the blue channel (excitation wavelength: 405 nm) and 515–550 nm for the green channel (excitation wavelength: 488 nm). Scale bar: 20 µm.

The biological applicability of BPN-NBD imaging of biothiols in a living system was conducted as encouraged by the excellent spectroscopic performance of BPN-NBD in aqueous media. Prior to fluorescence imaging, cytotoxicity of the BPN-NBD to living MCF-7 cells was checked by MTT staining method (Supplementary Figure S14). The related results revealed that different concentrations (5.0, 10.0, 20.0, 50.0, 100.0 μM) of BPN-NBD all displayed stable high levels of cell survival rate (over 80%) after 24 h in MCF-7 cells, which suggested that BPN-NBD had negligible toxicity and good biocompatibility to the live-cell imaging. Then, the imaging results in living MCF-7 cells were obtained. As shown in Figure 4, living MCF-7 cells were incubated in BPN-NBD (10.0 μM) medium for 30 min at room temperature. Owing to the merits of MCF-7 cell membrane permeability for BPN-NBD, endogenous biothiols were detected with obvious fluorescence signals observed in blue and green channels (Figures 4A1,A2). As a sharp contrast, the treatment of MCF-7 cells pre-incubated with thiol-blocking reagent (1.0 mM for N-ethylmaleimide, NEM) induced a result of no fluorescence response under the same conditions (Figures 4B1,B2). The subsequent experiments as envisaged, after adding Cys or Hcy for 30 min and BPN-NBD for an additional 30 min, the NEM-treated MCF-7 cells send out remarkable fluorescence signals in blue (Figures 4C1,C2) and green channels (Figures 4D1,D2). When the NEM-treated MCF-7 cells were co-incubated with GSH and then BPN-NBD successively (Figures 4E1,E2), an obvious fluorescence signal was researched only in the blue channel rather than in two channels.

### Imaging of Thiols in Zebrafish

**FIGURE 5:**
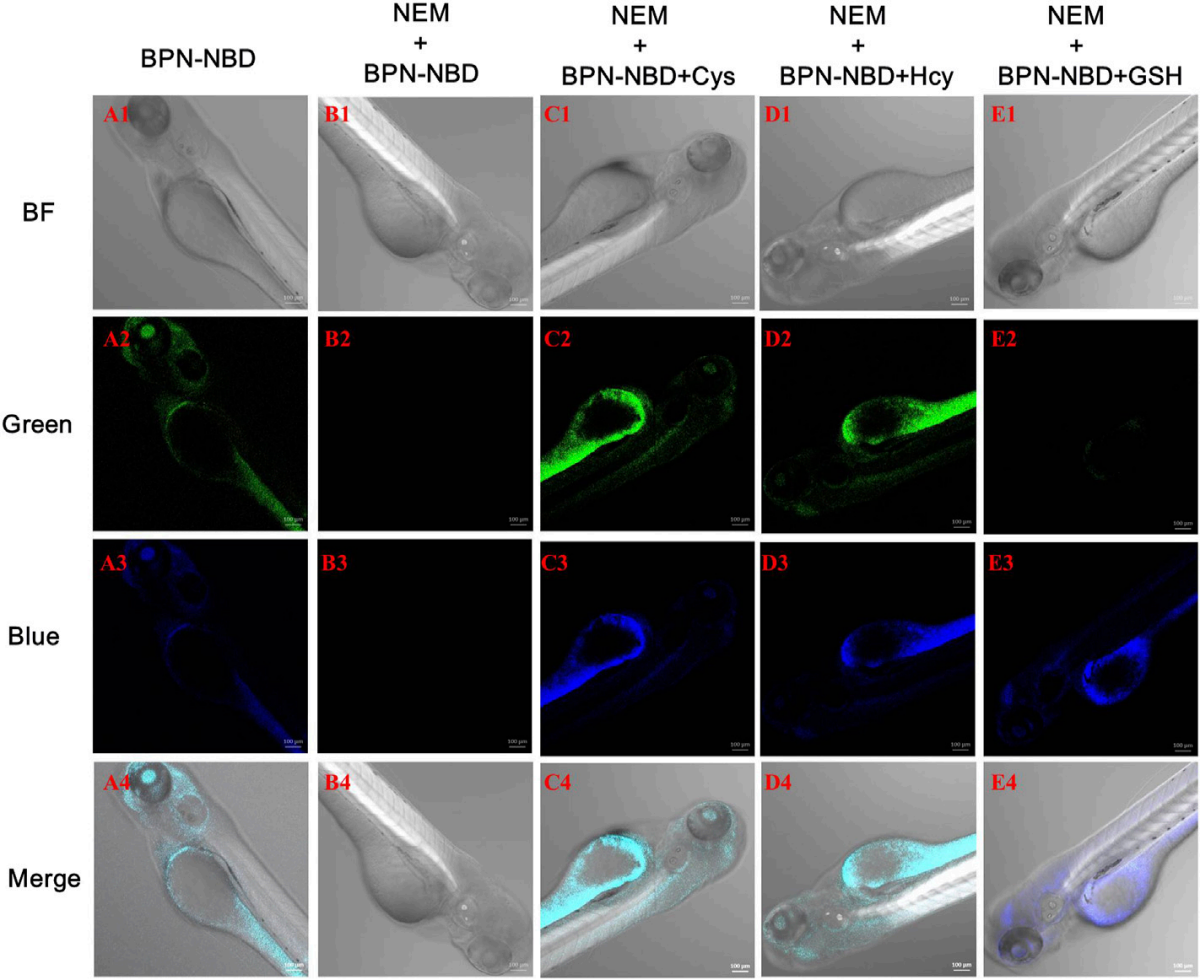
The images of BPN-NBD responding to biothiols in zebrafish. **(A)** Zebrafish incubated with BPN-NBD (10.0 µM) alone for 30 min. **(B)** Zebrafish treated with NEM (1.0 mM) and then incubated with BPN-NBD (10.0 µM) for 30 min **(C–E)** Pre-treated NEM zebrafish incubated with (150.0 µM Cys/Hcy or GSH, respectively) for 30 min and then treated with BPN-NBD (10.0 µM) for another 30 min. The confocal imagings (bright-field and fluorescence at green/blue channel) were collected. Emissions were collected at 425–475 nm for the blue channel (excitation wavelength: 405 nm) and 515–550 nm for the green channel (excitation wavelength: 488 nm). Scale bar: 100 µm.

We sought to evaluate the ability for Cys/Hcy and GSH detection of BPN-NBD in living zebrafish because of the promising performance of BPN-NBD in MCF-7 cells. As shown in Figure 5, zebrafish pre-incubated with BPN-NBD (10.0 μM) for 30 min displayed intense fluorescence in green (Figure 5A2) and blue channels (Figure 5A3). However, NEM-loaded zebrafish treated with BPN-NBD (10.0 μM) did not exhibit fluorescence emission in green (Figure 5B2) and blue channels (Figure 5B3). Next, when zebrafish were pretreated with NEM for 30 min, treated with Cys/Hcy (150.0 μM) for 30 min, and treated with BPN-NBD (10.0 μM) for 30 min, distinct strong fluorescence signals were collected from green (Figures 5C2,D2) and blue channels (Figures 5C2,D2). With regard to GSH (150.0 μM), strong fluorescence was observed in the blue channel (Figure 5E2) and no fluorescence was found in the green channel (Figure 5E3). These phenomena indicated that BPN-NBD could detect Cys/Hcy and GSH in zebrafish through a dual-emission signal manner.

### Imaging of Thiols in Tumor Tissue

**FIGURE 6:**
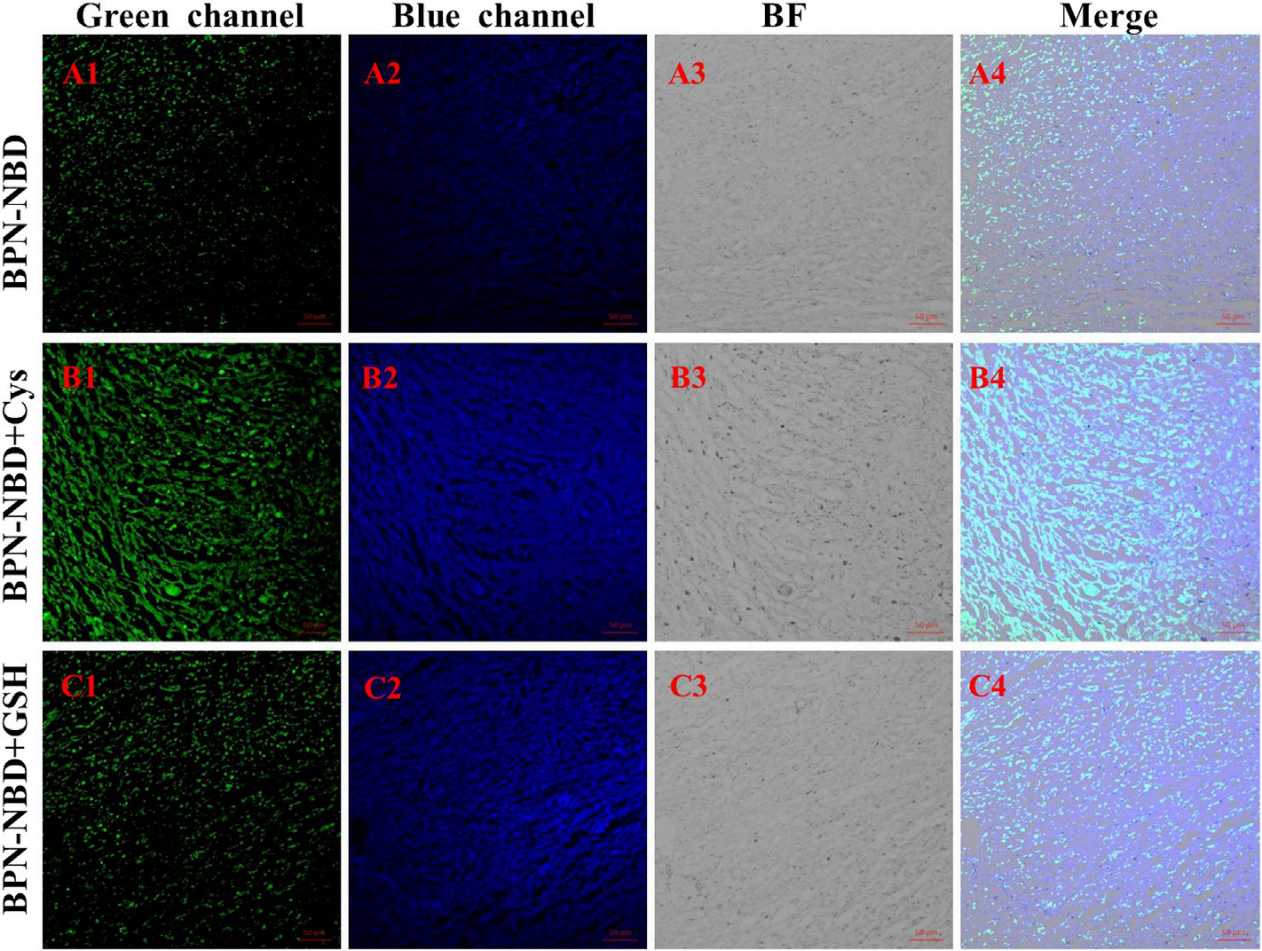
The tumor tissue imaging (at the green and blue channels) of endogenous and exogenous Cys/GSH in BALB/c mice. **(A)** Only with treatment by BPN-NBD (50.0 µM). **(B)** By staining with Cys (200.0 µM) and BPN-NBD (50.0 µM). **(C)** By dealing with GSH (200.0 µM) and BPN-NBD (50.0 µM). Emissions were collected at 425–475 nm for the blue channel (excitation wavelength: 405 nm) and 515–550 nm for the green channel (excitation wavelength: 488 nm). Scale bar: 50 µm.

Probe BPN-NBD for imaging endogenous and exogenous Cys/GSH in 4T1 breast cancer cell inoculated 4-week-old female BALB/c mice was evaluated. As displayed in Figure 6, we found green (Figure 6A1) and blue (Figure 6A2) fluorescences when tumor tissues in female BALB/c mice were injected and stained with 50.0 μM BPN-NBD for 1.5 h. As expected, the apparent fluorescence enhancement in green (Figure 6B1) and blue (Figure 6B2) channels were detected by treating the tumor tissues in female BALB/c mice with 200.0 μM Cys and 50.0 μM BPN-NBD for 1.5 h. Then, tumor tissues in female BALB/c mice treated with 200.0 μM GSH and 50.0 μM BPN-NBD for 1.5 h showed a pronounced fluorescence enhancement only in the blue channel (Figure 6C2). The outstanding performance of BPN-NBD towards Cys/GSH detection in tumor tissues indicated that BPN-NBD had great potential for clinical application in cancer diagnosis.

## Conclusion

In conclusion, by combining 4′-hydroxy-[1,1′-biphenyl]-4-carbonitrile (BPN-OH, fluorophore 1) with NBD as recognition site and fluorophore 2 by a facile ether bond, a simple fluorescent probe, BPN-NBD, was constructed for effective discrimination of Cys/Hcy and GSH with dual-emission signals via a single-wavelength excitation. BPN-NBD presented green and blue emissions in the presence of Cys/Hcy, and BPN-NBD responded to GSH by only displaying blue emissions. In addition, BPN-NBD exhibited excellent sensitivity for quantitative analysis toward Cys/Hcy and GSH. It also displayed easy-to-synthesize properties, rapid detection ability, high selectivity, and low toxicity, and it could work via a single-wavelength excitation. Furthermore, BPN-NBD was successfully applied in imaging of Cys/Hcy from GSH in living MCF-7 cells, tumor tissues, and zebrafish through a dual-emission signal manner. Overall, we believe that BPN-NBD had great potential for clinical application in cancer diagnosis.

## Data Availability

The original contributions presented in the study are included in the article/Supplementary Material, further inquiries can be directed to the corresponding author.
