# Supplementary material for: Simultaneous Discrimination of Cys/Hcy and GSH With Simple Fluorescent Probe Under a Single-Wavelength Excitation and its Application in Living Cells, Tumor Tissues, and Zebrafish
Source: Front Chem. 2022 Mar 11;10:856994. doi: 10.3389/fchem.2022.856994 (PMC8961673; doi:10.3389/fchem.2022.856994)
Supplement: Supplementary file 1 [file DataSheet1.PDF]

Supporting information for

Simultaneous discrimination of Cys/Hcy and GSH with simple fluorescent probe under a single-wavelength excitation and its application in living cells, tumor tissues, and zebrafish

Dongling Yan [1,†], Likun Liu [2,†], Xiangbao Liu[1], Qi Liu[2], Peng Hou[1], Hao Wang[1], Chunhui Xia[1], Gang Li[2], Chunhui Ma[1] and Song Chen[1,*]

[1] College of Pharmacy, Qiqihar Medical University, Qiqihar, 161006, P. R. China

[2] Research Institute of Medicine & Pharmacy, Qiqihar Medical University, Qiqihar, 161006, P. R. China

[†]D. Y. and L. L. contributed equally to this work

[*]Corresponding author, E-mail address: chensongchemistry@163.com

Table of contents

Page

TableS1...S1

Figures S1-2.. S3

Figures S3-4.. S4

Figures S5-6.. S5

Figures S7-8.. S6

Figures S9-10.. S7

Figures S11-12.. S8

Figures S13-14.. S9

Figures S15-16.. S10

Figures S17...S11

Table S1. The reported **NBD**-based fluorescent probes for simultaneous discrimination of Cys/Hcy and GSH.

Probe	Excitation mode	Test system	Detection limit	Response time	Application	Literature
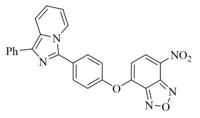	Double wavelength	PBS buffer with 30% EtOH	140 nM for Cys, 180 nM for Hcy, 78 nM for GSH	6 min	HepG2 cells	Dyes and Pigments, 2021, 191, 109381
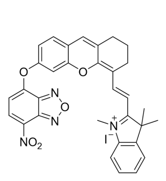	Double wavelength	PBS buffer	0.027 μM for Cys, 0.025 μM for Hcy, 0.016 μM for GSH	20 min for Cys, 20 min for Hcy, 5 min for GSH	HeLa cells	Biosensors and Bioelectronics, 2016, 81, 341-348
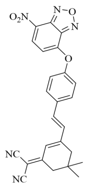	Single wavelength	HEPES with 50% ethanol	0.051μM for Cys; 0.016μM for Hcy; 0.034μM for GSH	10 min	L929 cells	Dyes and Pigments, 2017, 140, 212-221
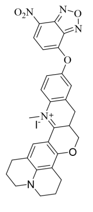	Single wavelength	PBS buffer	0.012μM for Cys; 0.013μM for Hcy; 0.06 μM for GSH	15 min for Cys; 28 min for Hcy; 15 min for GSH	HeLa cells	Sensors and Actuators B-Chemical, 2018, 273, 1170-1178
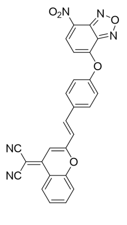	Double wavelength	PBS buffer with 50% DMSO	0.021 μM for Cys, 0.017 μM for Hcy, 0.026 μM for GSH	15 min for Cys, 15 min for Hcy, 10 min for GSH	MCF-7 cells Nude mice	Sensors and Actuators B Chemical, 2017, 245, 297-304
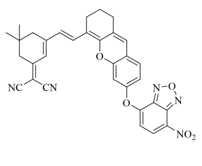	Double wavelength	PBS buffer with 40% DMF	0.165 μM for Cys, 0.106μM for Hcy, 0.555 μM for GSH -	8 min	HeLa cells	Dyes and Pigments, 2021, 186, 109015

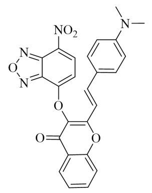	Single wavelength	PBS buffer with 30% CH_3CN	0.021 μM for Cys; 0.027 μM for Hcy; 0.064 μM for GSH	60 min	HeLa cells	Anal. Chem., 2016, 88, 3638-3646
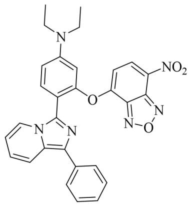	Double wavelength	PBS buffer with 50% DMSO	21.4 nM for Cys; 46.2 nM for Hcy; 63.5 nM for GSH	2.5 min	A549 cells Tumor issues	New J. Chem., 2018, 42, 18172--18181
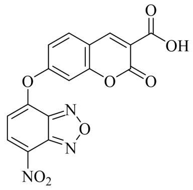	Double wavelength	PBS buffer with 20% DMSO	4.92 μM for Cys; 0.046 μM for Hcy; 0.295 μM for GSH	-------	-------	Journal of Fluorescence 2021, 31,599–607
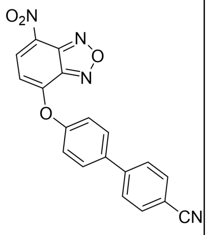	**Single wavelength**	**PBS buffer with 1 mM CTAB**	**0.011 μM for Cys, 0.015 μM for Hcy, 0.003 μM for GSH**	**150 s**	**MCF-7 cells Tumor issues zebrafish**	**This work**

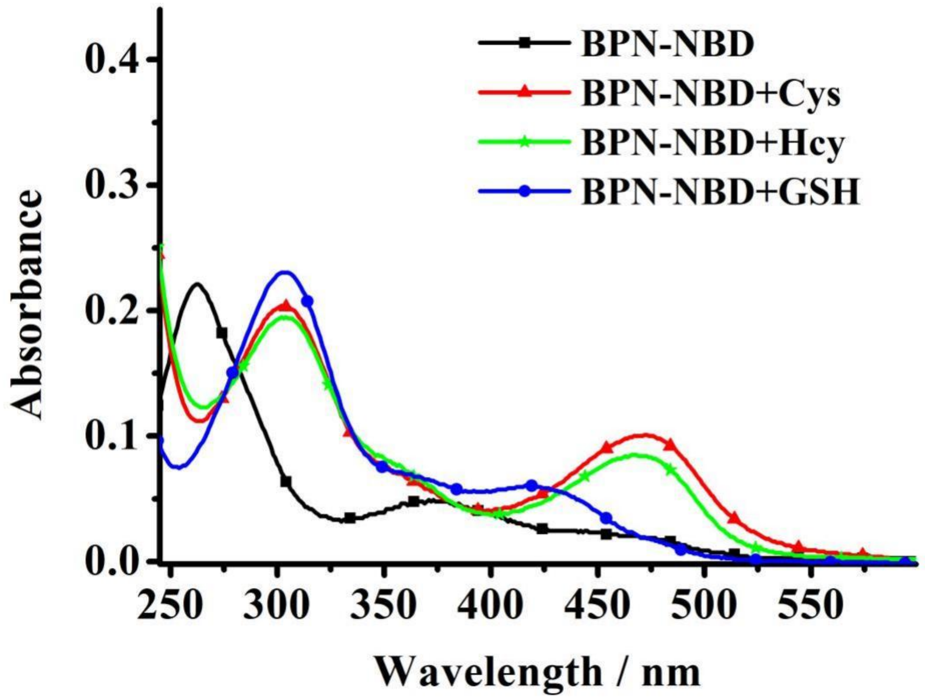

Fig.S1 The absorption spectra of probe **BPN-NBD** (black line) and **BPN-NBD** reacted with Cys (red line), Hcy (green line), GSH (blue line).

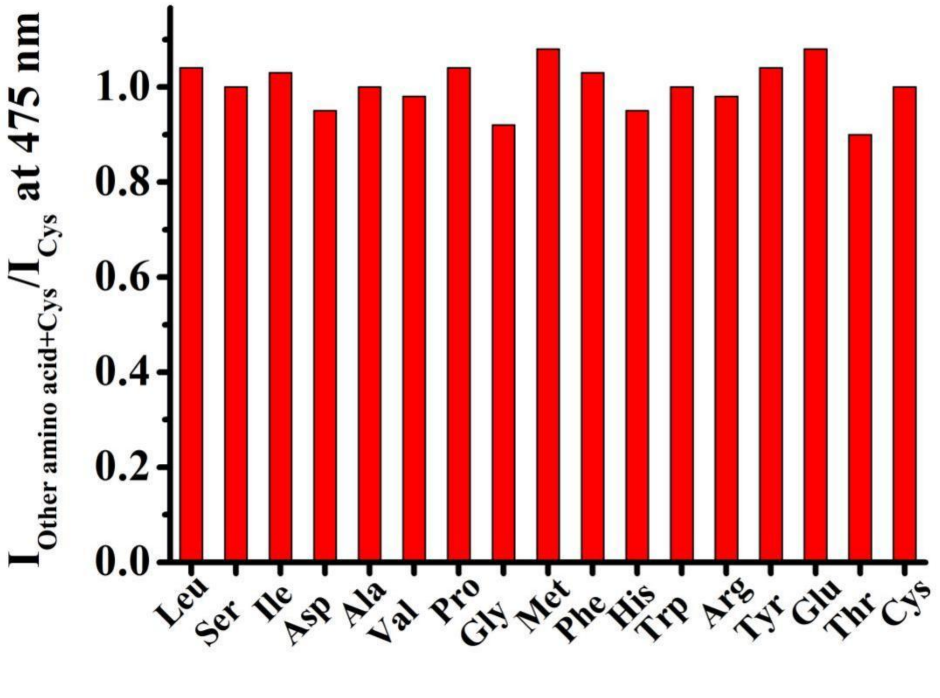

Fig.S2 Fluorescence response of **BPN-NBD** (10.0 μM) towards Cys (50.0 μM) with

the competition other amino acids (100.0 μM) at 475 nm.

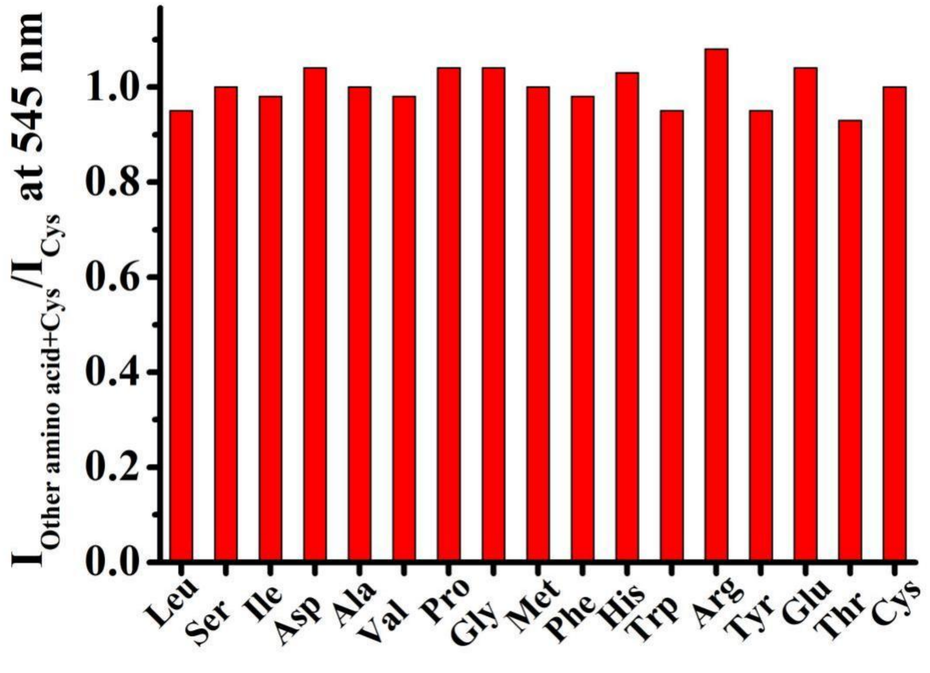

Fig.S3 Fluorescence response of **BP**N-NBD (10.0 μM) towards Cys (50.0 μM) with the competition other amino acids (100.0 μM) at 545 nm.

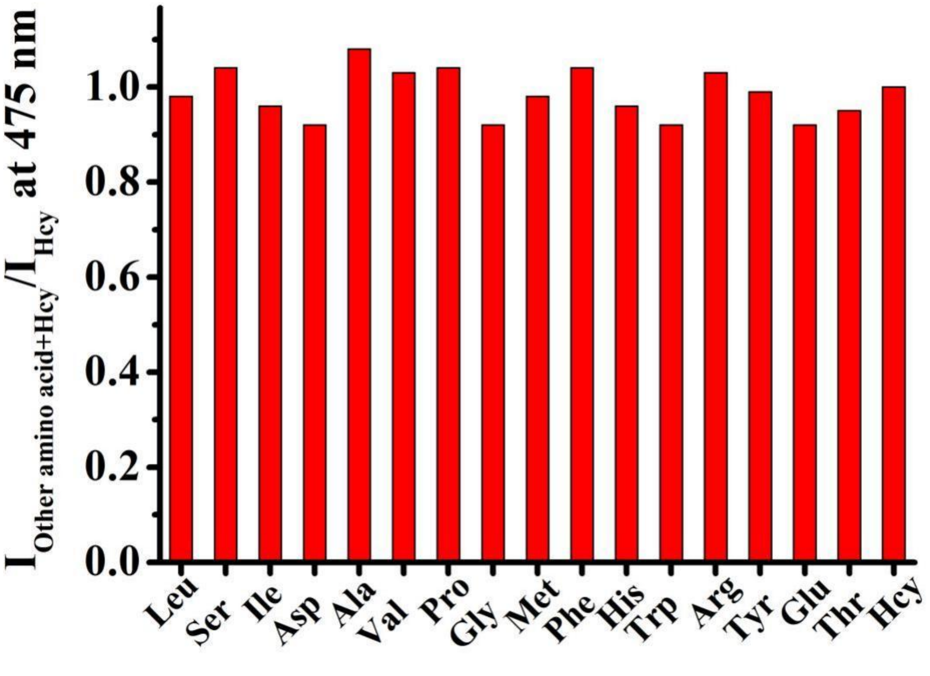

Fig.S4 Fluorescence response of **BPN-NBD** (10.0 μM) towards Hcy (50.0 μM) with the competition other amino acids (100.0 μM) at 475 nm.

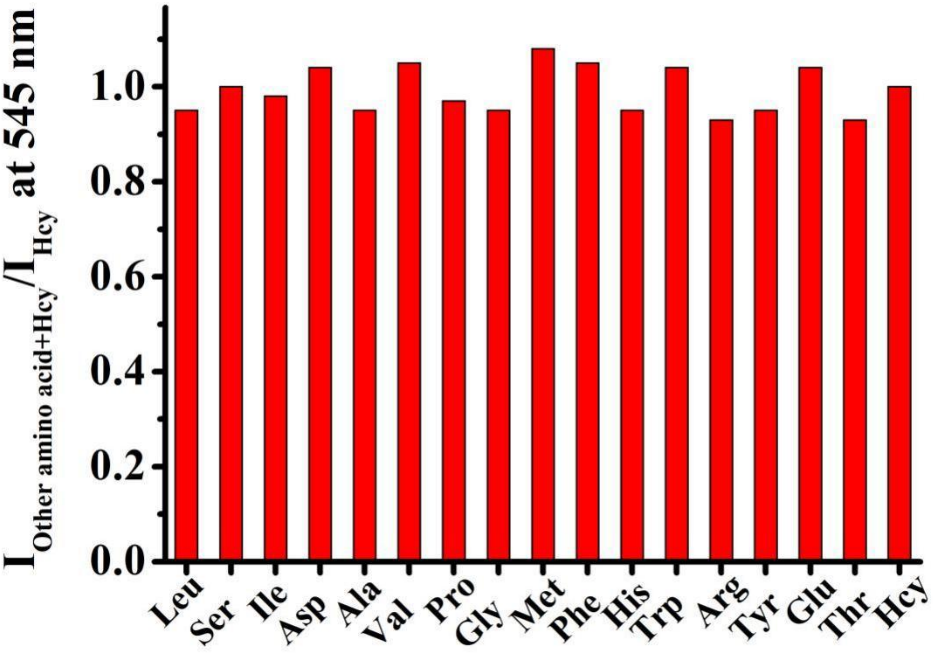

Fig.S5 Fluorescence response of **BPN-NBD** (10.0 μM) towards Hcy (50.0 μM) with the competition other amino acids (100.0 μM) at 545 nm.

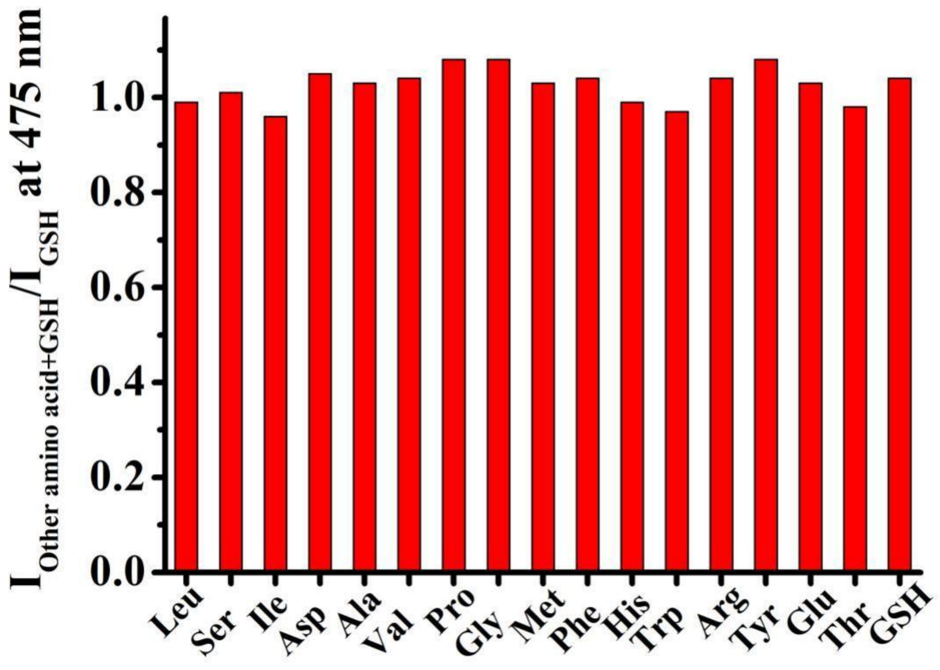

Fig.S6 Fluorescence response of **BPN-NBD** (10.0 μM) towards GSH (50.0 μM) with the competition other amino acids (100.0 μM) at 475 nm.

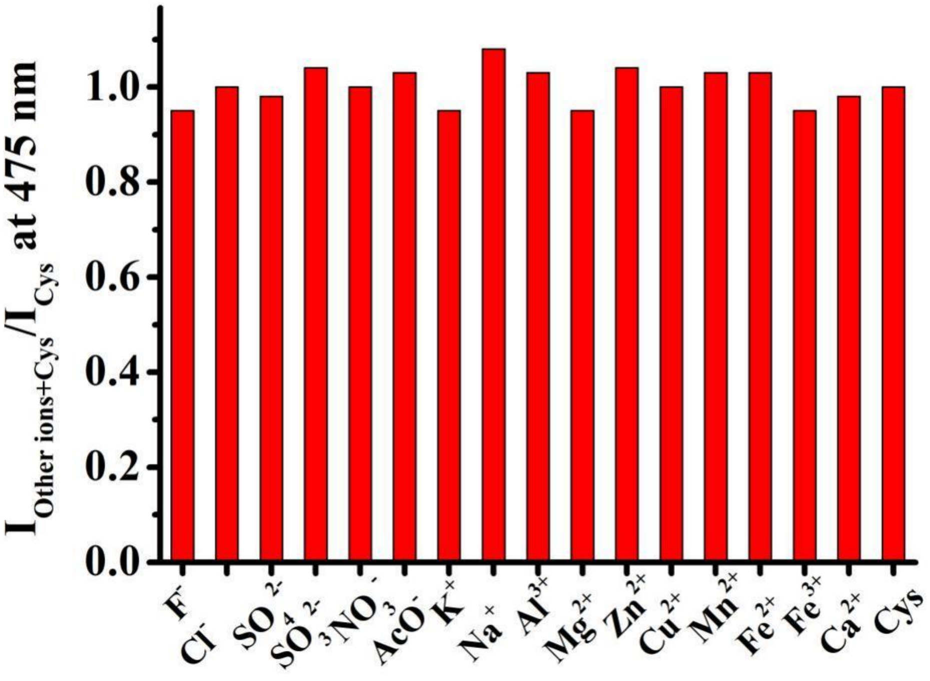

Fig.S7 Fluorescence response of **BPN-NBD** (10.0 μM) towards Cys (50.0 μM) with the competition ions (500.0 μM) at 475 nm.

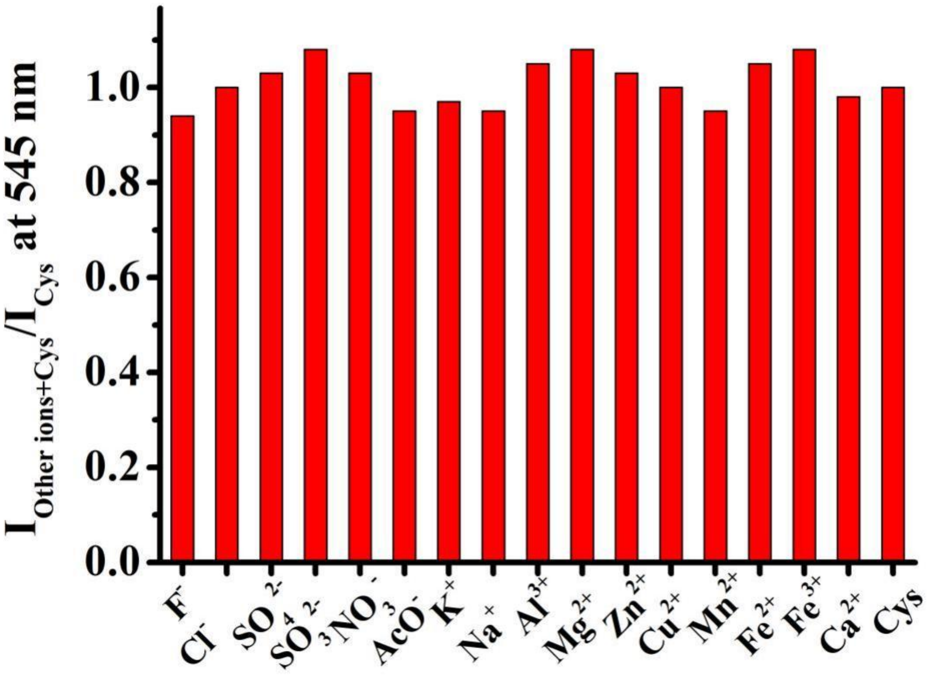

Fig.S8 Fluorescence response of **BPN-NBD** (10.0 μM) towards Cys (50.0 μM) with the competition ions (500.0 μM) at 545 nm

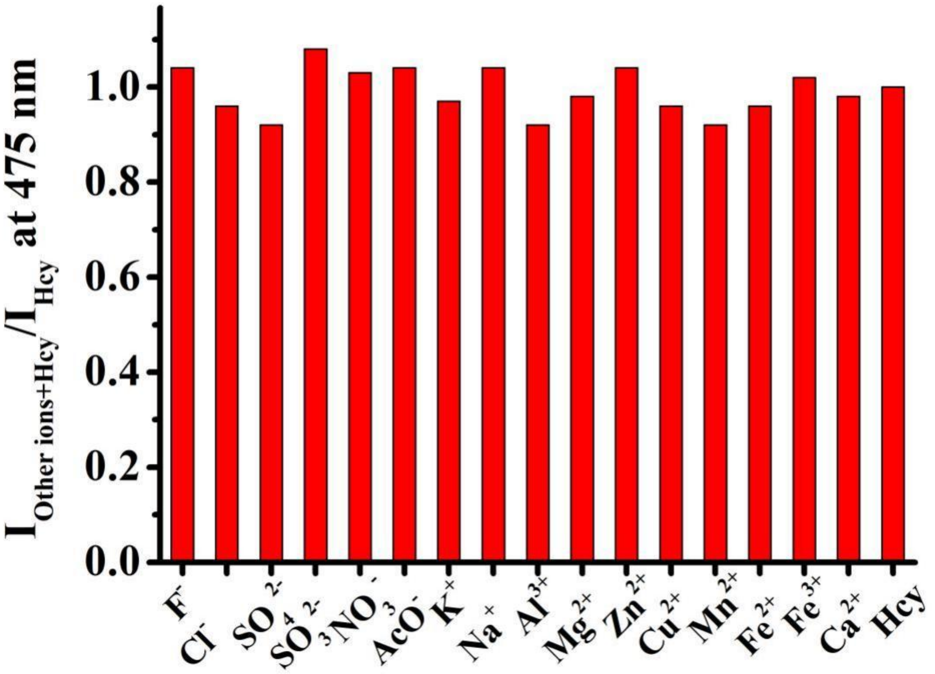

Fig.S9 Fluorescence response of **BPN-NBD** (10.0 μM) towards Hcy (50.0 μM) with the competition ions (500.0 μM) at 475 nm.

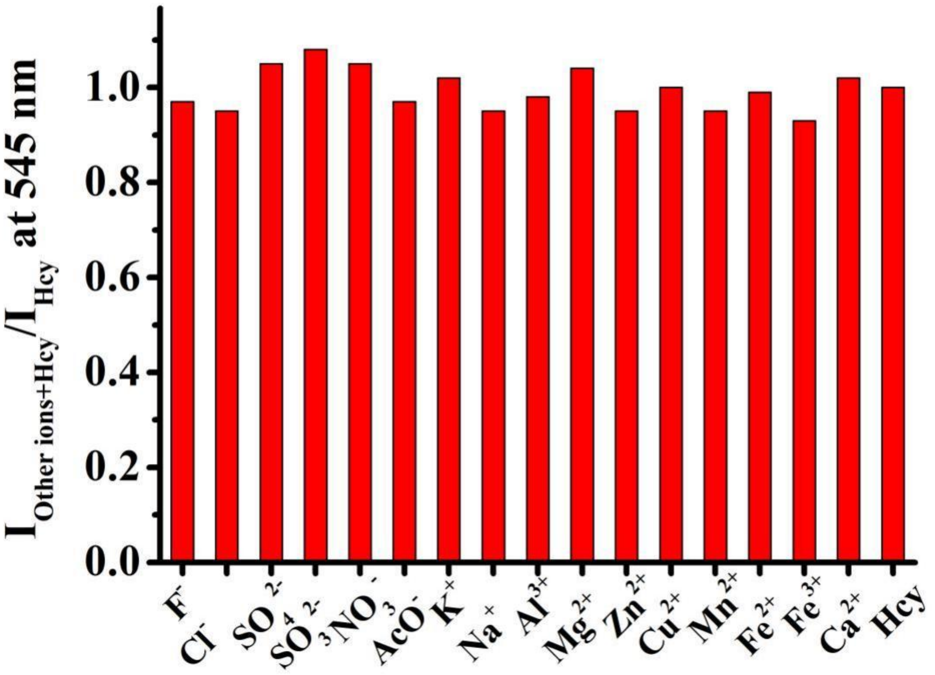

Fig.S10 Fluorescence response of **BPN-NBD** (10.0 μM) towards Hcy (50.0 μM) with the competition ions (500.0 μM) at 545 nm.

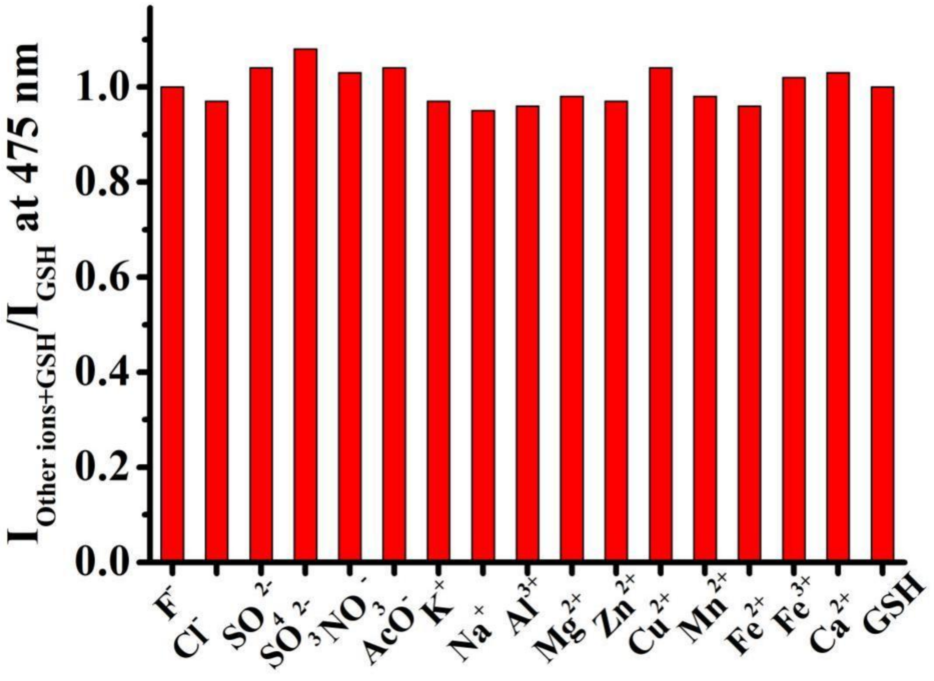

Fig.S11 Fluorescence response of **BPN-NBD** (10.0 μM) towards GSH (50.0 μM) with the competition ions (500.0 μM) at 475 nm.

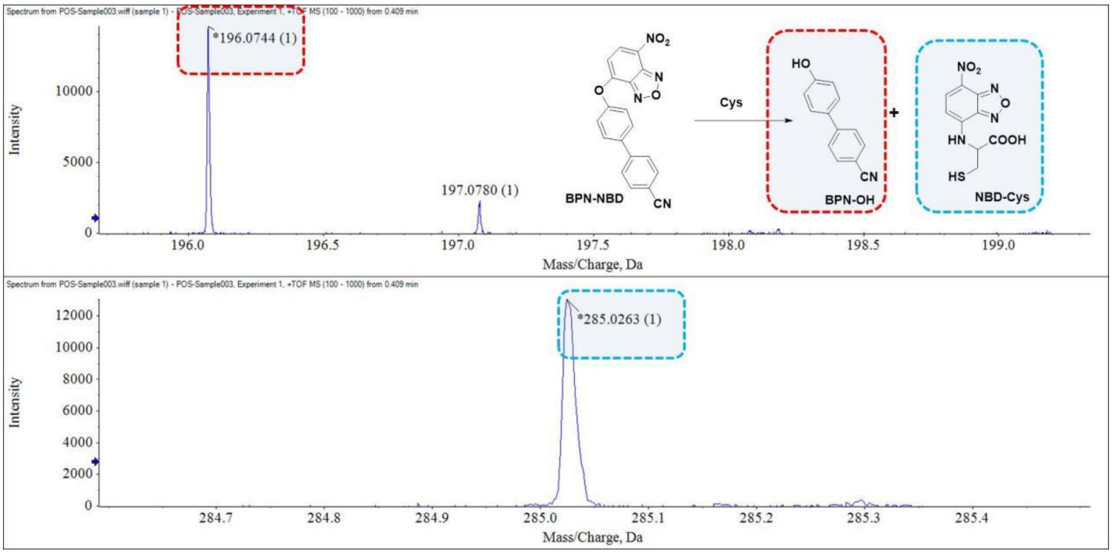

Fig.S12 HRMS spectrum of **BPN-NBD** + Cys.

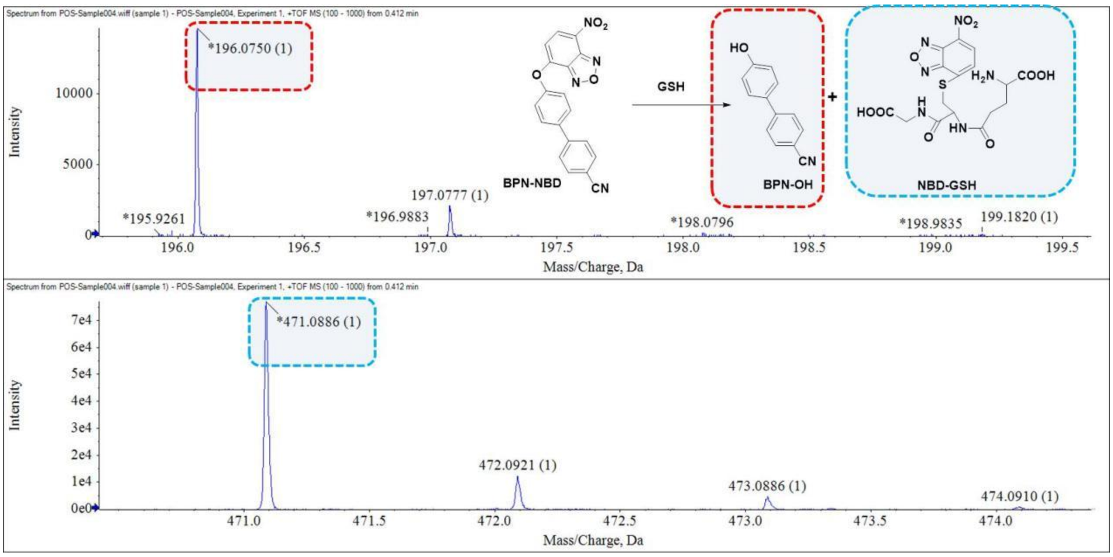

Fig.S13 HRMS spectrum of **BPN-NBD** + GSH.

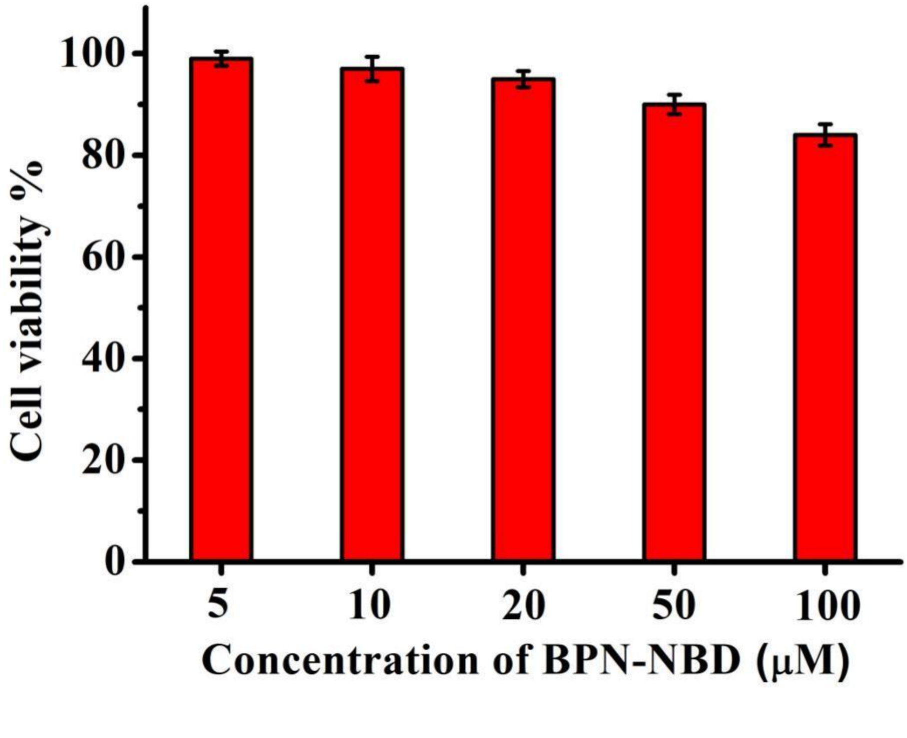

Fig.S14 Percentage of viable MCF-7 cells after treatment with indicated concentrations (5.0, 10.0, 20.0, 50.0, 100.0 μM) of **BPN-NBD** after 24 hours.

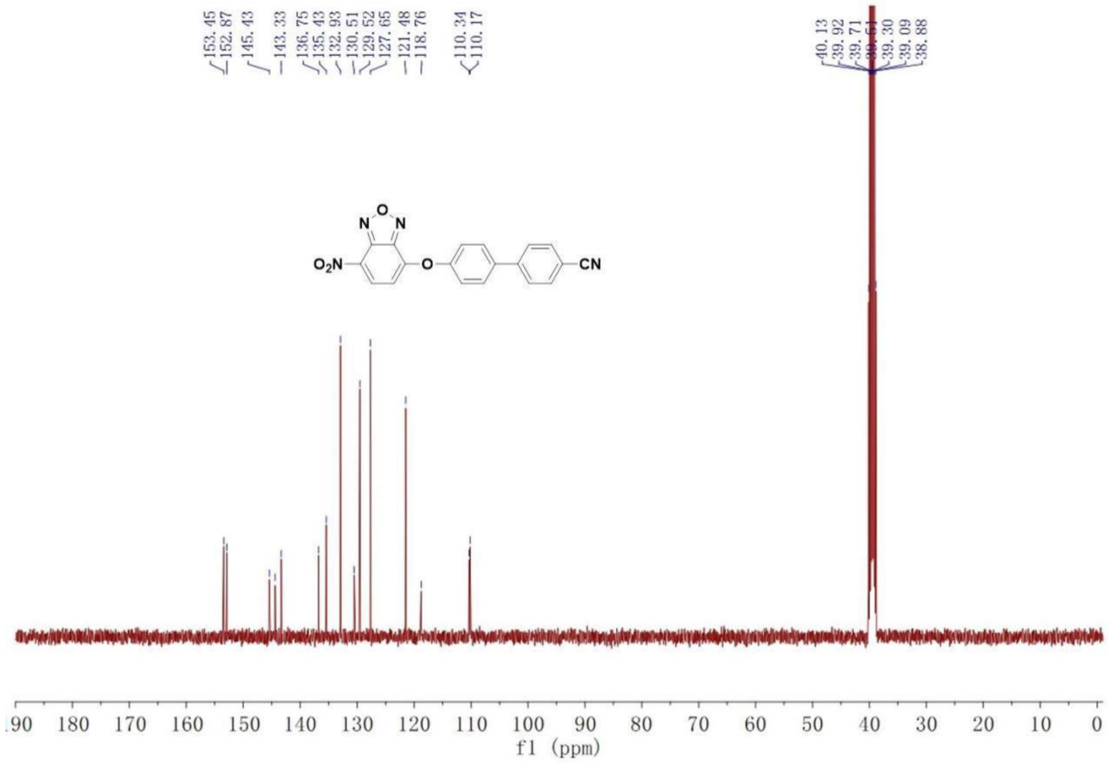

Fig.S15 ^{13}C NMR spectrum of **BPN-NBD** in DMSO-d_6.

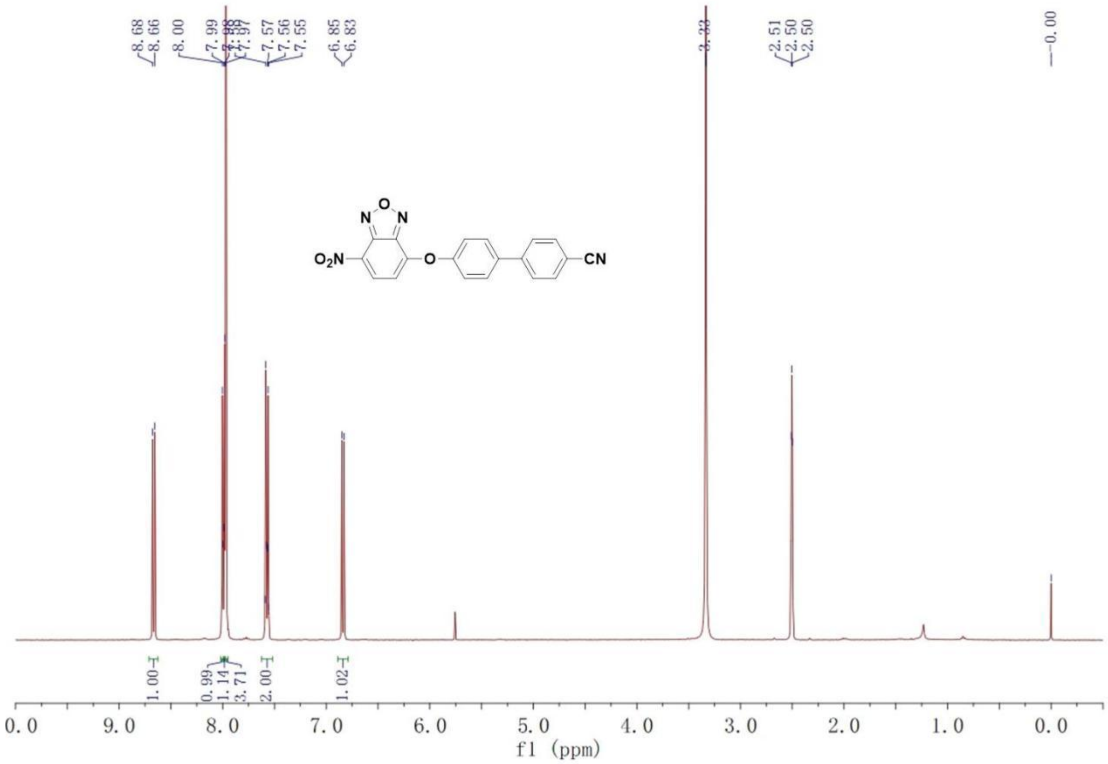

Fig.S16 ^{1}H NMR spectrum of **BPN-NBD** in DMSO-d_6.

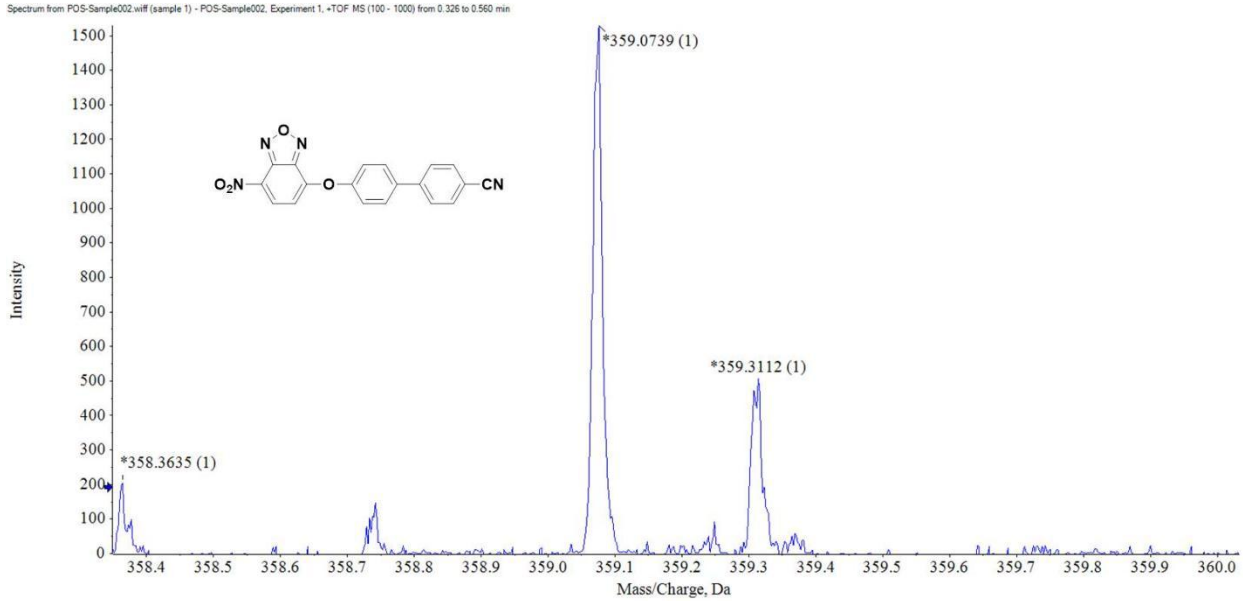

Fig.S17 HRMS spectrum of **BPN-NBD**.
